# Draft genome sequence of *Staphylococcus gallinarum* BAU_KME002 strain isolated from egg surface in Bangladesh

**DOI:** 10.1128/MRA.00555-23

**Published:** 2023-09-22

**Authors:** Fatimah Muhammad Ballah, Md. Saiful Islam, Md. Liton Rana, Mst. Minara Khatun, Marzia Rahman, Jayedul Hassan, Md. Tanvir Rahman

**Affiliations:** 1 Department of Microbiology and Hygiene, Faculty of Veterinary Science, Bangladesh Agricultural University, Mymensingh, Bangladesh; Portland State University, Portland, Oregon, USA

**Keywords:** whole-genome sequencing, *S. gallinarum*, poultry, antibiotic resistance, virulence factors, Bangladesh

## Abstract

This report describes the genome sequence of the *Staphylococcus gallinarum* BAU_KME002 strain isolated in Bangladesh in 2021 from a chicken egg surface. Our assembled genome had 50 contigs, an estimated genome length of 2,866,882 bp (with coverage of 90.0×), 36 predicted antibiotic resistance genes, and 28 predicted virulence factor genes.

## ANNOUNCEMENT

*Staphylococcus gallinarum* is commonly found in the environment and is primarily documented in poultry (1). While *S. gallinarum* is typically not considered pathogenic in humans, it has been found in infected wounds of hospitalized individuals, the blood of a patient with chronic hepatitis B infection (2), and in cases of eye infection, specifically endophthalmitis (3).

From June 2021 to March 2022, we collected egg surface swab samples from various poultry farms and egg markets located in the Mymensingh district of Bangladesh (24.7539°N, 90.4073°E). These samples were then transported to our laboratory (24.7196°N, 90.4267°E) and subjected to overnight incubation at 37°C in nutrient broth (HiMedia, India). Following incubation, the samples were streaked onto Mannitol Salt agar (HiMedia, India) plates, and the resulting colonies underwent staining and biochemical tests to isolate *S. gallinarum* (4). The matrix-assisted laser desorption ionization time-of-flight mass spectrometry was employed to identify *S. gallinarum* (5). Finally, a *S. gallinarum* isolate was aerobically grown on a 5% bovine blood agar plate, followed by incubating at 37°C for 24 h. The Qiagen DNA Mini Kit (QIAGEN, Hilden, Germany) was then utilized to extract the genomic DNA of *S. gallinarum* BAU_KME002 from the broth culture. Next, a sequencing library was generated by employing the Nextera DNA Flex Library Prep Kit (Illumina, San Diego, CA, USA), and it was subsequently sequenced on the Illumina NextSeq2000 platform using paired-end reads (2  ×  150). The Unicycler.v0.4.9 (6) tool was employed to assemble the genome, and the raw paired-end reads (*n* = 14,246,490) were trimmed using Trimmomatic.v0.39 (7). The quality of the trimmed reads was assessed using FastQC.v0.11.7 (8). Subsequently, the genome was annotated using Prokka.v1.14.6 (9), PATRIC.v3.2.76 (10), and PGAP.v3.0 (11). The presence of antibiotic resistance genes (ARGs) was determined using CARD.v3.2.4 (12), NDARO.v2023 (13), and PATRIC.v3.2.76 (10), while virulence factor genes (VFGs) were identified using VFDB (14) and Victors (15). PathogenFinder.v2.0 (16) was utilized to assess the pathogenicity index, and DrugBank.v4.0 (17) and TTD (18) were referenced for drug target genes, TCDB (19) for transporter genes, and RAST.v2.0 (20) for metabolic functional features in the assembled genome. Default parameters were used for all software unless otherwise specified.

Our assembled *S. gallinarum* BAU_KME002 genome consisted of 50 contigs, 4 L50 contigs with an N50 value of 184,051 bp, a total length of 2,862,301 bp, an average G + C content of 33.16%, and 61 RNA genes. The general characteristics of the *S. gallinarum* BAU_KME002 are documented in Table 1.

**TABLE 1:** General characteristic features of the *Staphylococcus gallinarum* BAU_KME002 strain

Attributes	Values
Genome size	2,862,301 bp
Genome coverage	90.0×
G + C content	33.16
Contig L50	4
Contig N50	184,051 bp
Total genes	2,785
Coding sequences	2,762
Coding genes	2,689
Protein coding genes	2,689
RNA genes	61
tRNA genes	4
rRNAs	57
Pseudo genes	31
Hypothetical proteins	616
Proteins with functional assignments	2,146
Genes assigned to SEED subsystems	1,249
Number of subsystems	279

**Fig 1:**
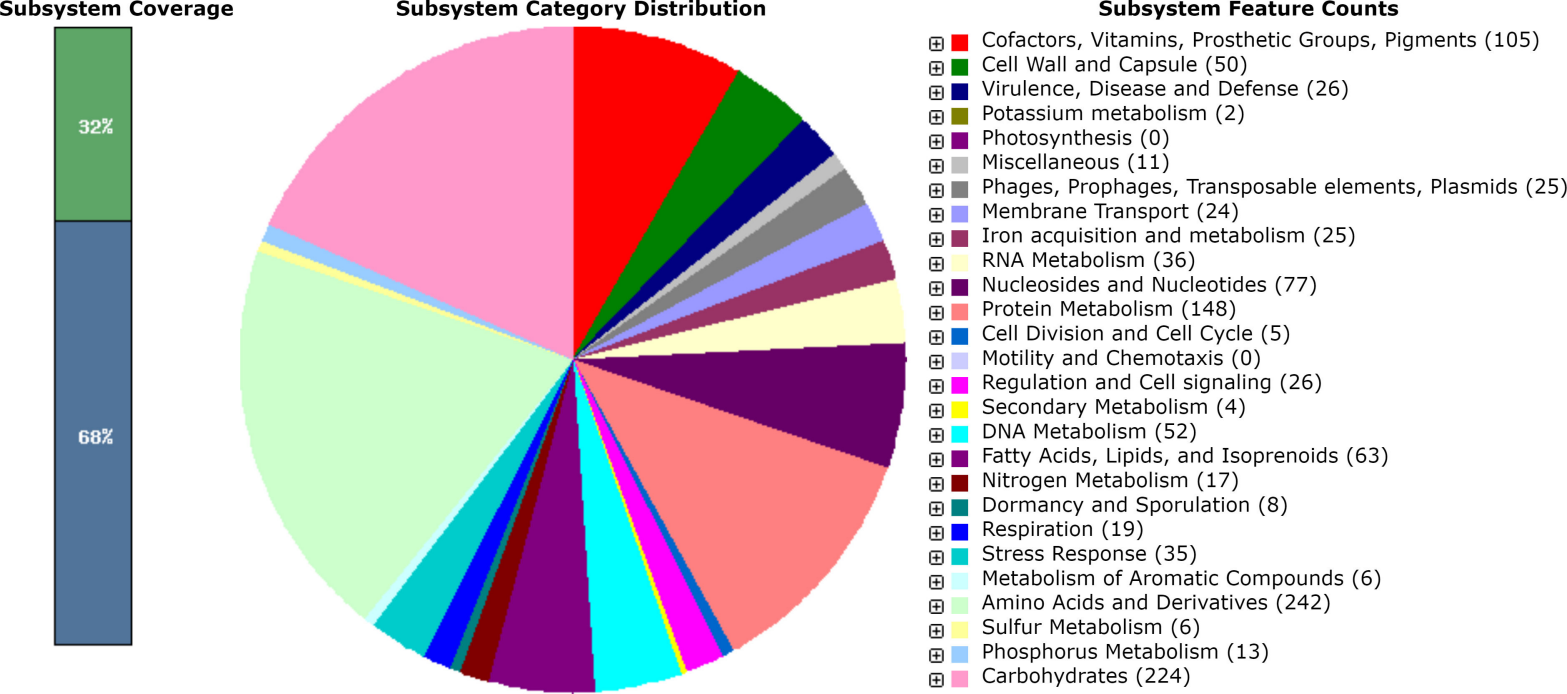
Metabolic functional features in the assembled *Staphylococcus gallinarum* BAU_KME002 genome.

In PathogenFinder, our genome exhibits a probability of pathogenicity index of 0.981 (98.1%) towards a human host. The *S. gallinarum* BAU_KME002 genomes harbored 38 predicted ARGs under various antibiotic categories. Our annotated genome had 15 predicted drug target genes and 17 predicted transporter genes. In addition, the current genome contained 28 predicted VFGs under different virulence determinants. Moreover, our genome harbored 279 subsystems with 32% coverage and 1,249 genes (Table 1; Fig. 1).

## Data Availability

The WGS shotgun analysis of *S. gallinarum* BAU_KME002 was submitted to GenBank under the accession number JAPQEW000000000. The relevant data, including the raw reads, were deposited with BioProject accession number PRJNA907246, BioSample accession number SAMN31957329, and SRA accession number SRR22509343. In this paper, the specific version referred to is JAPQEW000000000.1.
